# Tree seedlings respond to both light and soil nutrients in a Patagonian evergreen-deciduous forest

**DOI:** 10.1371/journal.pone.0188686

**Published:** 2017-11-30

**Authors:** Alvaro Promis, Robert B. Allen

**Affiliations:** 1 Department of Silviculture and Nature Conservancy, University of Chile, Santiago, Chile; 2 Independent Researcher, Lincoln, New Zealand; Universite du Quebec a Chicoutimi, CANADA

## Abstract

Seedlings of co-occurring species vary in their response to resource availability and this has implications for the conservation and management of forests. Differential shade-tolerance is thought to influence seedling performance in mixed *Nothofagus betuloides*–*Nothofagus pumilio* forests of Patagonia. However, these species also vary in their soil nutrient requirements. To determine the effects of light and soil nutrient resources on small seedlings we examined responses to an experimental reduction in canopy tree root competition through root trenching and restricting soil nutrient depletion through the addition of fertilizer. To understand the effect of light these treatments were undertaken in small canopy gaps and nearby beneath undisturbed canopy with lower light levels. Seedling diameter growth was greater for *N*. *pumilio* and height growth was greater for *N*. *betuloides*. Overall, diameter and height growth were greater in canopy gaps than beneath undisturbed canopy. Such growths were also greater with fertilizer and root trenching treatments, even beneath undisturbed canopy. Seedling survival was lower under such treatments, potentially reflecting thinning facilitated by resource induced growth. Finally, above-ground biomass did not vary among species although the less shade tolerant *N*. *pumilio* had higher below-ground biomass and root to shoot biomass ratio than the more shade tolerant *N*. *betuloides*. Above- and below-ground biomass were higher in canopy gaps so that the root to shoot biomass ratio was similar to that beneath undisturbed canopy. Above-ground biomass was also higher with fertilizer and root trenching treatments and that lowered the root to shoot biomass ratio. Restricting soil nutrient depletion allowed seedlings of both species to focus their responses above-ground. Our results support a view that soil nutrient resources, as well as the more commonly studied light resources, are important to seedlings of *Nothofagus* species occurring on infertile soils.

## Introduction

The resources required for seedling development and growth are often scarce in forests [1–2]. The creation of canopy gaps can generate temporary increases in forest understory resources such as light, soil nutrients, and/or moisture [3–4]. Just how seedlings respond to these increased resources is known to vary with species-specific traits [5–6]. One trait commonly related to seedling light responses is shade tolerance [5, 7]. However, where both light and soil nutrient are enhanced by canopy gap creation it is challenging to distinguish the effects of shade tolerance from the effects of nutrient demand [5–6]. Yet, we must determine how both light and soil nutrients influence seedlings if we are to develop a mechanistic understanding of tree regeneration processes. Effective management of forests for sustainable timber production, alleviating the effects of invasive species, or mitigating the effects of climate change on forest structure and composition require an understanding of these processes [8].

A framework has emerged for how the seedlings of different tree species might respond to changes in light and soil nutrients [5, 9]. It has been postulated that more shade-tolerant species are less likely to exhibit a positive growth response to increased soil nutrients under high light conditions than less shade-tolerant species [5]. This is because less shade-tolerant species tend to be more adaptable to changing light [7] and are therefore more able to respond to an increase in both resources [6]. The availability of soil nutrients can also moderate the light requirements of less shade-tolerant species [10] so that increased nutrient availability can also allow such species to grow more rapidly under lower light conditions. Increased soil nutrients and light may not only increase seedling growth but also survivorship [3, 10]. What is more, changes in light and soil nutrient resources can impact on how seedlings allocate biomass above- and below-ground [5, 9, 11]. Increased soil nutrient availability can reduce the root to shoot biomass ratio of seedlings due to increased shoot growth to capture light [11–12].

In this paper, we determine the effect of light and soil nutrients on naturally occurring seedlings in a mixed *Nothofagus betuloides* (Mirb.) Oerst.–*Nothofagus pumilio* (Poepp. & Endl.) Krasser forest on Tierra del Fuego, Chile. These forests are transitional between the evergreen *N*. *betuloides* stands that occur to the west on higher rainfall sites with less fertile soils and those on more fertile soils to the east with lower rainfall where the deciduous *N*. *pumilio* occurs [13–14]. The former is considered more shade-tolerant than the latter [15–16]. We examine responses by seedlings of both species to enhanced soil nutrient availability through the experimental addition of fertilizer and reduction of canopy tree root competition through root trenching. Fertilizer addition restricts nutrient resource depletion while trenching more clearly links to the removal of canopy tree root access and inhibition [5, 17–18]. To understand the influence of light, these manipulations were implemented beneath relatively undisturbed canopies and in nearby naturally formed canopy gaps. We expect seedling growth will be greater in canopy gaps. We hypothesize that trenching and fertilization will increase *N*. *pumilio* seedling growth in canopy gaps above that of the more shade tolerant *N*. *betuloides* [5]. We also expect that where increased resources lead to greater growth by seedlings there will also be higher survivorship [10]. As an evergreen, *N*. *betuloides* is adapted to low soil nutrient conditions and might also maintain a higher root to shoot biomass ratio than *N*. *pumilio*. We hypothesize that fertilizer addition and root trenching would reduce the root to shoot biomass ratio of both species, but particularly *N*. *pumilio*, due to increased shoot growth [11–12]. Alternatively, seedlings can increase below-ground allocation, when subjected to increased soil nutrients, to maximize short-term uptake.

## Materials and methods

### Study area

The study sampled an uneven-aged *N*. *betuloides–N*. *pumilio* stand that has not been logged for timber. This stand is located within the Río Bueno Research Station (53° 45´ S, 69° 58´ W; 90–140 m above sea level), Karukinka Natural Park, on the western Chilean side of Tierra del Fuego. The 16.8 ha stand had a density of 730 trees ha^-1^ and basal area of 96.2 m^2^ ha^-1^. *N*. *betuloides* trees dominate the stand with 89.2% of the density and 80.2% of the basal area. The seedlings of both species were young and of similar age (3 to 10 years). Mixed stands of these two species occur between 40°S and 55°S latitude in southern Chile and Argentina [15].

The stand is in an area that is part of the subalpine zone in glacially formed U-shaped valleys [19]. During the warmest month, the mean air temperature ranges between 9.0 and 9.5°C and remains above 0.0°C in the coldest month. The annual precipitation is approximately 500–600 mm. Maximum wind speed can exceed 100 km h^−1^ [20]. The podzolized soils are typically shallow (< 50 cm), loamy in texture, acidic (pH 4.1–5.5), infertile and maintain high rooting levels in the litter layer [13–14]. The high C:N ratio in the soil organic horizons of *N*. *betuloides* dominated forests likely results in immobilization of N [14].

Treefall-induced canopy gaps in mixed *N*. *betuloides–N*. *pumilio* forests in Tierra del Fuego range between 21 and 898 m^2^; most are <100 m^2^ [16]. The common gap-makers are uprooted and wind-snapped trees. Transmitted solar radiation is greater in these small canopy gaps than beneath undisturbed canopies. Such radiation is particularly low in undisturbed canopies to the north of canopy gaps because of a low sun angle in these high latitude forests [21].

### Experimental design

A systematic search of the stand in January 2011 identified a total of 36 recently formed canopy gaps with areas ranging between 27 and 564 m^2^. Such canopy gaps were identified when the horizontal projection of a canopy opening to the forest floor was larger than 20 m^2^ and the downed trees still retained their bark. Eight of these canopy gaps, with areas ranging between 45 and 190 m^2^, were randomly selected for this experimental study.

**Fig 1:**
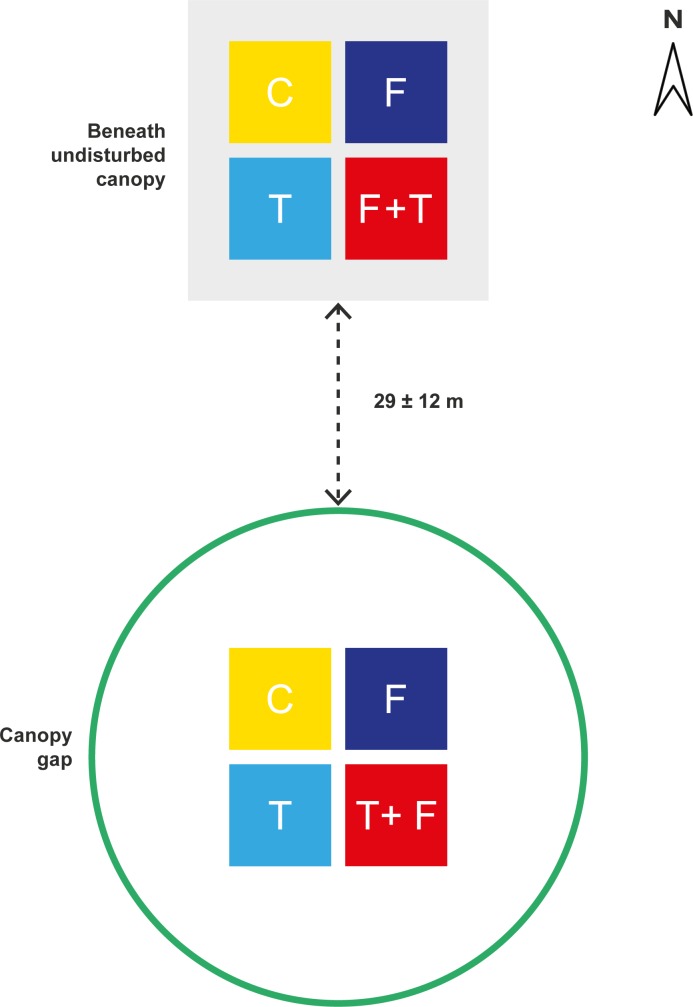
Factorial design of the experiment undertaken on seedlings of two co-occurring species under two canopy conditions. The canopy gap is outlined by a green circle and beneath undisturbed canopy as a shaded area. The layout of four 2 × 2 m plots are shown as colored squares within each of the canopy conditions. C = control; F = fertilizer; T = root trenching; F+T = fertilizer+root trenching. The design shown was replicated eight times and the mean distance (± SD) to the north (N) between canopy gap plots and beneath undisturbed canopy plots is given.

The experiment had a factorial design with responses by seedlings of the two species measured within treated plots under two canopy conditions (Fig 1). Four adjacent 4 m^2^ plots (2 × 2 m) were established in the center of each of the eight canopy gaps. Because the seedlings studied in these plots were of a similar size they all would have been subjected to increased solar radiation. The same arrangement of 4 m^2^ plots was also established beneath undisturbed canopy to the north of each canopy gap, with the distance large enough to overcome light reaching the undisturbed canopy plots from the gaps [21]. Canopy openness was measured at the center of each plot using a canopy-scope [22]. The canopy-scope consists of a transparent plastic sheet marked with a grid of 5 × 5 dots, spaced at intervals of 3 cm, and held 20 cm from the eye. Readings with these scopes are strongly correlated with values of canopy openness estimated from hemispherical photographs [22]. Percent canopy openness was calculated as the ratio of the dots in canopy gaps to the total number of dots. Four readings were obtained with the scope at each plot by facing each of the cardinal directions (North, West, South and East) and averaged.

One of three treatments (fertilizer (F), root trenching (T) and fertilizer+root trenching (F+T)) was randomly applied to a plot in each canopy condition (canopy gap and beneath undisturbed canopy). The fourth plot in each canopy condition was a control (Fig 1). A multi-purpose fertilizer was applied to plots at the beginning of the first growing season (Table 1). We applied such a fertilizer because previous work has not shown which mineral nutrients are most limiting, although N and P were likely candidates. The rationale for fertilizer application considered the ratio among macro-nutrients and their absolute amounts. The ratio used was that typically required to meet seedling needs [23]. The absolute amount applied used quantities previously shown to generate productivity responses in high elevation New Zealand *Nothofagus* forests [17, 24]. It is worth noting that fertilizer addition can also increase the rate of litter decomposition and further increase the availability of nutrients [25].

**Table 1:** Fertilizer component compounds and quantity applied to each plot in November 2011. Also given are the nutrient contributed by each compound and the per unit area nutrient equivalent.

Compound	Quantity (g)	Nutrient	Equivalent (kg ha^-1^)
Urea	348	N	400
Triple super phosphate	45	P	52
Sulphate potash magnesia	89	Mg	40
Lime	8	Ca	40
Muriate of potash	21	K	80

At the same time as fertilizer application the boundary of root trenched plots was cut to 30 cm depth using a spade to sever roots entering each seedling plot. As all canopy trees were rooted outside seedling plots, trenching severed the roots of canopy trees growing into plots. Trenching also severed roots from seedlings located just outside the plots although the boundary was of a sufficient distance from the plot edge that seedlings were not affected within plots. Root trenching removed most below-ground competition with canopy trees because *Nothofagus* have high rooting density in the organic horizon (<10 cm depth) and are at times confined to this horizon [14]. Severing the canopy tree roots not only restricts root access and the uptake of nutrients but also removes the ability of roots from canopy trees to interfere with and inhibit the development of seedling roots [18]. While severing the canopy tree roots may create an input of nutrients from the decomposing roots [26] other authors consider these are unlikely to be of significance to seedling growth compared with the large effects of removing below-ground competition of neighboring plants [27]. At the beginning and end of each subsequent growing season the trench around each plot was recut.

### Data collection

In each plot, all naturally occurring *N*. *betuloides* and *N*. *pumilio* seedling were tagged and the height to the top bud and root collar diameter (RCD) were measured in November 2011. In March 2013, after two austral growing seasons height and RCD of each live tagged seedling were re-measured. At this final measurement each seedling was excavated by hand and divided into leaves, branches, stem and roots. All parts of each seedling were dried in a forced air oven (70°C). Leaf mass, branch mass, stem mass, total above-ground biomass (the sum of leaf, branch and stem masses), and below-ground root biomass were determined.

The initial measurement was used to calculate seedling density and the plot level mean RCD and height. The remeasurement of tagged seedlings was used to calculate RCD growth (DG) and height growth (HG). These growth parameters were calculated as the mean of all individual seedlings, by species, alive at the end of the experiment in each plot. In addition, for each plot, tagged seedling density at the beginning and end of the experiment were used to calculate seedling survivorship. The final root to shoot biomass ratio (R:S ratio) of each seedling was calculated as root biomass/above-ground biomass based upon dry weight and the mean calculated for seedlings on each plot.

### Statistical analysis

Plots were used as the experimental unit in each analysis. Two-way ANOVA was used to test whether initial canopy openness varied with canopy condition and treatment. Three-way ANOVA was used to test for effects of species, canopy condition and treatment on mean initial seedling density, height, and RCD as well as survivorship, and final biomass allocation. Biomass allocation was assessed as mean seedling above-ground biomass (A-GB; leaf, branches and stem), below-ground biomass (B-GB; root), and R:S ratio. Three-way ANCOVA was used to test for effects of species, canopy condition and treatment on mean DG and HG. Species, canopy condition and treatment were all analyzed as fixed effects. Comparisons were made using Tukey’s HSD test as a post-hoc test [28]. To reduce heteroscedasticity and to improve normality, initial seedling density, DG, HG, A-GB, B-GB and R:S ratio were ln-transformed prior to statistical analyses. The percentage canopy openness and percentage of seedlings surviving were arcsine-square root transformed [28]. All statistical analyses were carried out using SPSS 15.0 for Windows (SPSS, Inc.). All differences shown in the Results were significant at 5% level.

## Results

### Initial conditions

**Fig 2:**
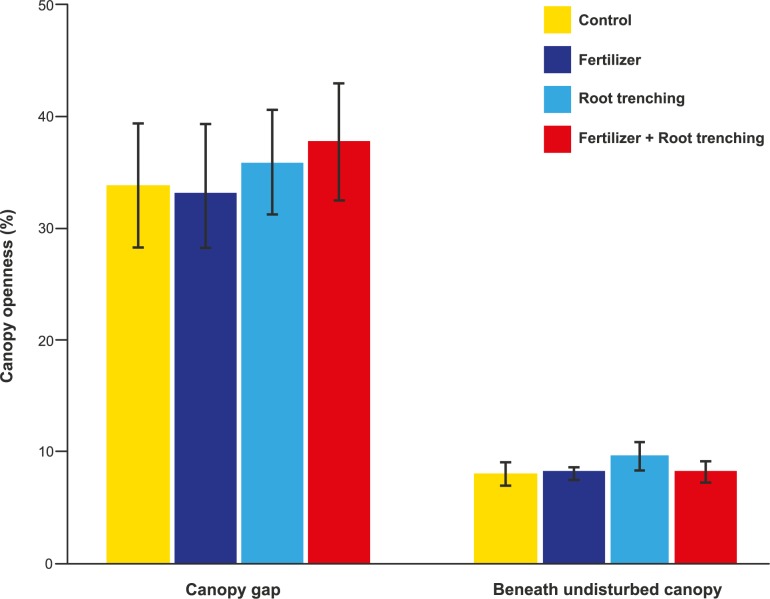
Mean canopy openness (%) with respect to canopy condition (i.e. canopy gap and beneath undisturbed canopy), control (C) and three treatments (F = fertilizer, T = root trenching, F+T = fertilizer+root trenching). Error bars represent ± 1 SE.

Mean canopy openness in canopy gaps (35.3 ± 2.4; mean ± SE) was four times that beneath undisturbed canopy (8.6 ± 0.5; F = 108.04, p < 0.001). There was no difference in canopy openness for control plots versus plots subjected to fertilizer addition and root trenching treatments (F = 0.132, p = 0.941; Fig 2; S1 Table).

Initial seedling density did not vary with species, canopy condition, or treatment (Table 2; S2 Table). The initial RCD of *N*. *pumilio* was 32% greater than that of *N*. *betuloides*, but did not vary with canopy condition or treatment. The initial seedling heights of *N*. *pumilio* were slightly less than those of *N*. *betuloides*, but did not vary with canopy condition or treatment (Table 2; S2 Table). There were no species, canopy condition, or treatment interactions for seedling density, initial root collar diameter and initial height.

**Table 2:** Three-way ANOVA for mean (± SE) initial density (D), initial root collar diameter (RCD) and initial height (H) of seedlings per plot by species, canopy condition and treatment as main factors.

Factor	Source of variation	D (seedlings m^-2^)	RCD (mm)	H (mm)
Species	Nb	5.4 ± 0.58	0.73 ± 0.03	42.7 ± 0.88
	Np	5.2 ± 0.49	0.96 ± 0.02	39.8 ± 0.94
	*F (p)*	*0*.*007 (0*.*934)*	*43*.*262 (0*.000)*	*5*.*022 (0*.027)*
Condition	CG	5.1 ± 0.48	0.85 ± 0.03	41.7 ± 0.93
	BUC	5.5 ± 0.59	0.83 ± 0.03	40.9 ± 0.92
	*F (p)*	*0*.*001(0*.*970)*	*0*.*454 (0*.*502)*	*0*.*371 (0*.*544)*
Treatment	C	4.7 ± 0.60	0.83 ± 0.05	41.9 ± 1.32
	F	4.4 ± 0.60	0.87 ± 0.04	43.3 ± 1.53
	T	5.9 ± 0.73	0.84 ± 0.04	40.0 ± 1.07
	F+T	6.2 ± 1.01	0.81 ± 0.04	39.9 ± 1.22
	*F (p)*	*1*.*272 (0*.*288)*	*0*.*508 (0*.*678)*	*1*.*650 (0*.*182)*
*Interactions*				
*Species × Condition*	*F (p)*	*1*.*895 (0*.*171)*	*1*.*703 (0*.*195)*	*0*.*072 (0*.*789)*
*Species × Treatment*	*F (p)*	*0*.*804 (0*.*494)*	*0*.*153 (0*.*927)*	*0*.*225 (0*.*879)*
*Condition × Treatment*	*F (p)*	*0*.*784 (0*.*505)*	*0*.*785 (0*.*505)*	*2*.*222 (0*.*090)*
*Species × Condition × Treatment*	*F (p)*	*0*.*876 (0*.*456)*	*0*.*650 (0*.*585)*	*0*.*507 (0*.*679)*

Nb, *Nothofagus betuloides*; Np, *Nothofagus pumilio*; CG, canopy gap; BUC, beneath undisturbed canopy; C, control; F, fertilizer; T, root trenching; F+T, fertilizer+root trenching; F, Fisher test; (p) = probability.

* = significant differences at p < 0.05.

### Seedling growth

DG was higher for *N*. *pumilio* than *N*. *betuloides* and higher in canopy gaps than beneath undisturbed canopy. F or F+T increased such growth above that on control plots (Table 3; S3 Table). In contrast, HG was higher for *N*. *betuloides* than *N*. *pumilio*. Such growth was 27% greater in canopy gaps than beneath undisturbed canopy, and 52% greater in the F+T treatment than controls (Table 3; S3 Table). There were no species, canopy condition, or treatment interactions for seedling growth.

**Table 3:** Three-way ANCOVA for mean (± SE) diameter growth (DG) and height growth (HG) of seedlings per plot by species, canopy condition and treatment as main factors.

Factor	Source of variation	DG	HG
Species	Nb	0.65 ± 0.08	21.03 ± 1.51
	Np	0.76 ± 0.03	14.02 ± 0.99
	*F (p)*	*17*.*369 (< 0*.001)*	*14*.*650 (< 0*.001)*
Condition	CG	0.81 ± 0.05	19.62 ± 1.42
	BUC	0.60 ± 0.03	15.42 ± 1.23
	*F (p)*	*18*.*627 (< 0*.001)*	*7*.*011 (0*.009)*
Treatment	C	0.61 ± 0.04 c	13.81 ± 1.23 c
	F	0.75 ± 0.06 ab	20.55 ± 2.35 ab
	T	0.61 ± 0.05 bc	14.48 ± 1.09 bc
	F + T	0.81 ± 0.07 a	20.95 ± 2.19 a
	*F (p)*	*3*.*194 (0*.027)*	*4*.*531 (0*.005)*
*Interactions*			
*Species × Condition*	*F (p)*	*0*.*123 (0*.*726)*	*0*.*002 (0*.*963)*
*Species × Treatment*	*F (p)*	*2*.*403 (0*.*072)*	*0*.*241 (0*.*868)*
*Condition × Treatment*	*F (p)*	*0*.*565 (0*.*639)*	*1*.*179 (0*.*321)*
*Species × Condition × Treatment*	*F (p)*	*0*.*169 (0*.*917)*	*1*.*293 (0*.*281)*

The covariate was mean RCD and height of seedlings per plot for DG and HG respectively. Variables were ln-transformed for statistical analyses. Only for the DG analysis was the covariate significant (F = 5.580, p < 0.020) and therefore influenced ln of mean DG.

Nb, *Nothofagus betuloides*; Np, *Nothofagus pumilio*; CG, canopy gap; BUC, beneath undisturbed canopy; C, control; F, fertilizer; T, root trenching; F+T, fertilizer+root trenching; F, Fisher test; (p) = probability. Different letters indicate statistical differences using Tukey test post-hoc (*: p < 0.05).

### Seedling survival

Survival of *N*. *betuloides* and *N*. *pumilio* seedlings, after two growing seasons, varied with treatment but not with species or canopy condition (Table 4; S4 Table). Seedling survival in the F+T treatment was lower than in the C, F, and T treatments). There were no species, canopy condition, or treatment interactions for seedling survival (Table 4; S4 Table).

**Table 4:** Three-way ANOVA for mean percent seedling survival per plot (± SE) by species, canopy condition and treatment as main factors.

Factor	Source of variation	Seedling survival (%)
Species	Nb	48.0 ± 3.1
	Np	42.6 ± 3.1
	*F (p)*	*1*.*690 (0*.*196)*
Condition	CG	43.2 ± 2.9
	BUC	47.5 ± 3.3
	*F (p)*	*0*.*876 (0*.*351)*
Treatment	C	53.0 ± 2.9 b
	F	46.8 ± 4.6 b
	T	50.1 ± 4.4 b
	F + T	31.4 ± 4.6 a
	*F (p)*	*5*.*085 (0*.002)*
*Interactions*		
*Species × Condition*	*F (p)*	*0*.*023 (0*.*850)*
*Species × Treatment*	*F (p)*	*0*.*997 (0*.*397)*
*Condition × Treatment*	*F (p)*	*2*.*634 (0*.*053)*
*Species × Condition × Treatment*	*F (p)*	*0*.*381 (0*.*767)*

Nb, *Nothofagus betuloides*; Np, *Nothofagus pumilio*; CG, canopy gap; BUC, beneath undisturbed canopy; C, control; F, fertilizer; T, root trenching; F+T, fertilizer+root trenching; F, Fisher test; (p) = probability. Different letters indicate statistical differences using Tukey test post-hoc (*: p < 0.05).

### Seedling biomass allocation

A-GB at the end of the experiment did not differ with species (Table 5; S5 Table). However, A-GB was influenced by canopy condition and treatment with A-GB 55% greater in canopy gaps than beneath undisturbed canopy and A-GB was 49% higher in the F treatment than the control. *N*. *pumilio* seedlings had higher B-GB than *N*. *betuloides* seedlings. B-GB was 53% higher in canopy gaps than beneath undisturbed canopy, while there was no effect of treatment on B-GB (Table 5; S5 Table). The R:S of *N*. *pumilio* seedlings was 33% higher than *N*. *betuloides* seedlings. The R:S ratio was not influenced by canopy condition, but was 24% smaller in the F + T treatment than controls. There were no species, canopy condition, or treatment interactions for A-GB, B-GB and R:S ratio (Table 5; S5 Table).

**Table 5:** Three-way ANOVA for mean (± SE) above-ground biomass (A-GB), below-ground biomass (B-GB) and root to shoot biomass ratio (R:S) of seedlings per plot by species, canopy condition and treatment as main factors.

Factor	Source of variation	A-GB (g)	B-GB (g)	R:S
Species	Nb	0.081 ± 0.007	0.046 ± 0.004	0.630 ± 0.026
	Np	0.067 ± 0.004	0.055 ± 0.004	0.841 ± 0.044
	*F (p)*	*2*.*852 (0*.*094)*	*4*.*302 (0*.040)*	*16*.*389 (0*.000)*
Condition	CG	0.090 ± 0.007	0.061 ± 0.004	0.773 ± 0.039
	BUC	0.058 ± 0.004	0.040 ± 0.004	0.698 ± 0.038
	*F (p)*	*22*.*560 (0*.000)*	*21*.*818 (0*.000)*	*2*.*350 (0*.*128)*
Treatment	C	0.061 ± 0.004 b	0.050 ± 0.006	0.854 ± 0.076 a
	F	0.091 ± 0.011 a	0.057 ± 0.007	0.676 ± 0.042 ab
	T	0.062 ± 0.005 ab	0.043 ± 0.003	0.757 ± 0.039 ab
	F + T	0.083 ± 0.009 ab	0.050 ± 0.006	0.645 ± 0.044 b
	*F (p)*	*3*.*711 (0*.014)*	*0*.*742 (0*.*529)*	*3*.*094 (0*.030)*
*Interactions*				
*Species × Condition*	*F (p)*	*0*.*038 (0*.*845)*	*0*.*269 (0*.*605)*	*0*.*528 (0*.*469)*
*Species × Treatment*	*F (p)*	*0*.*561 (0*.*642)*	*0*.*613 (0*.*608)*	*0*.*449 (0*.*719)*
*Condition × Treatment*	*F (p)*	*1*.*668 (0*.*178)*	*0*.*523 (0*.*667)*	*0*.*679 (0*.*567)*
*Species × Condition × Treatment*	*F (p)*	*0*.*351 (0*.*788)*	*0*.*505 (0*.*680)*	*0*.*707 (0*.*550)*

Nb, *Nothofagus betuloides*; Np, *Nothofagus pumilio*; CG, canopy gap; BUC, beneath undisturbed canopy; C, control; F, fertilizer; T, root trenching; F+T, fertilizer+root trenching; F, Fisher test; (p) = probability. Different letters indicate statistical differences using Tukey test post-hoc (*: p < 0.05).

## Discussion

Both Patagonian *Nothofagus* species are able to establish, survive and grow as seedlings in the shaded understory beneath undisturbed canopy and in canopy gaps [15–16, 29]. While *N*. *betuloides* was the dominant tree species in the stand, this was not reflected in initial seedling densities with both species having similar abundancies (Table 2). Veblen et al. [15] suggested that the more shade tolerant *N*. *betuloides* seedlings were favored in small canopy gaps and *N*. *pumilio* in larger canopy openings. As we sampled small canopy gaps, and beneath undisturbed canopy, it was therefore surprising that small seedlings of *N*. *betuloides* were not favored relative to *N*. *pumilio* (Table 2). Particularly as Cruz et al. [30] showed that when the overstory was dominated by *N*. *betuloides*, then seedlings of this species were also numerically dominant in Patagonian forests.

Small seedlings of both our *Nothofagus* species are released by canopy gaps and grow towards the main canopy [15]. As such we had expected there would be a difference in the initial seedling RCD or height between canopy gaps and undisturbed canopy, particularly for the less shade tolerant *N*. *pumilio* [15–16]. The absence of an initial size difference may partly reflect a short-term nutritional lag affecting growth. The canopy gaps studied were recent and nutrients likely remain sequestered in the relatively undecomposed fallen tree trunks that created the gaps [31–32]. Nitrogen can be immobilized in the early stage of woody debris decomposition and in so reduce soil N availability and seedling growth [33]. Alternatively, the lack of an initial seedling size difference may be because size poorly reflects previous growth.

### Seedling growth responses

RCD is widely considered a morphological indicator of seedling performance [34]. This indicator of performance held for DG, but did not hold for HG as *N*. *betuloides* seedlings grew faster than *N*. *pumilio* seedlings. This difference may reflect the allometries of each species. Another possibility was that selective browsing by guanaco (*Lama guanicoe*, Mueller 1776, Camelidae) restricted HG of *N*. *pumilio*. In the pure *N*. *pumilio* forests to the east browsing damage can be prevalent [35]. Although, in a pure *N*. *betuloides* forest, Promis et al. [29] showed that the level of guanaco browsing damage to seedlings was very low (< 3% of seedlings).

Our observation that seedlings of more shade-tolerant and less shade-tolerant tree species grow faster with higher light levels in canopy gaps than beneath closed canopies is similar to studies in both tropical [12] and temperate [5, 36] forests. Often it is the less shade tolerant species that exhibits the greatest increase with light [12, 36] although that was not so in our study because there was not an interaction between species and canopy condition (Table 3).

The lack of a significant interaction between canopy condition and treatment is the basis for rejecting our hypothesis that growth responses to F or T treatments would be greater in canopy gaps (Table 3). As such, our results are consistent with studies that show seedlings of both less shade-tolerant species [4, 12, 17] and more shade-tolerant species [12] increase growth after nutrient availability has been enhanced on infertile soils. That this occurred even beneath undisturbed canopies supports a view that the lack of light under closed canopies may not be restrictive if the soils are very infertile [5]. This is particularly important for small seedlings as their shallow root system makes them susceptible to below-ground competition on nutrient-poor soils [5]. DG and HG responses were greatest in relation to the F and F+T treatments (Table 3). As a consequence, we expect that restricting soil nutrient depletion is a stronger mechanistic explanation for seedling growth than root competition [18]. Our results contrast with a New Zealand study in mono-specific *Nothofagus* forest where relative diameter and height growth of seedlings growing beneath an undisturbed canopy on infertile soils were increased by root trenching, but not fertilizer addition [17]. The removal of canopy tree root access and inhibition by trenching may be more important for that New Zealand species because it has a dense shallow root network that rapidly proliferates to capture soil nutrients [17, 24].

### Seedling survivorship

Canopy gaps and root trenching increased the relative growth and survivorship of three tree species growing in a mesic, nutrient-rich temperate forest [4]. Others have found seedling survivorship to be higher with increasing soil resources in shaded environments [10, 37]. In contrast, our survivorship was least with treatments that had the greatest positive effect on growth (Tables 3 and 4). This may reflect, as proposed by Coomes and Grubb [5], intense competition for soil resources in the low fertility soils in our study area. Survivorship also tended to be less in canopy gaps where growth was also enhanced. Although this may not be so over a wider range of canopy covers [38]. An interspecific trade-off between high seedling survivorship under low-resource conditions (e.g. light or soil nutrients) versus high growth under high-resource conditions has been suggested in temperate and tropical forests [10, 36]. With no interactions between species, treatment, or canopy condition in our study we did not detect such a trade-off as both species demonstrated higher survivorship under lower resources and higher growth under higher resources.

### Seedling biomass allocation

We had expected that *N*. *betuloides* would maintain a higher R:S ratio than *N*. *pumilio* because it dominates forests on less fertile soils. Alternatively plants adapted to shade in the forest understory, like *N*. *betuloides*, can maintain a low R:S ratio [39]. It appears that *N*. *betuloides* conserves nutrients through its evergreen sclerophyllous leaf habit and the translocation of nutrients from absciissing leaves [40]. A relatively high below-ground allocation appears required by the less shade-tolerant *N*. *pumilio* to capture limiting soil resources. This greater below-ground allocation suggests the deciduous species allocates a greater root biomass to support leaf regrowth during the next growing season whereas the evergreen species require less because leaves are retained among years [41–42].

The R:S ratio of *N*. *pumilio* or *N*. *betuloides* were not affected by canopy condition (Table 5). This may be because seedlings attempt to capture both the elevated light and soil nutrients. This strategy was modified with F and F+T treatments where seedlings only increased A-GB and, in so doing, reduced the R:S ratio. The effect of increasing soil nutrients did not depend on species (no interactions in Table 5). Elsewhere it appears seedlings can increase below-ground allocation, when subjected to increased soil resources, to maximize short-term nutrient uptake [43].

### Implication for mixed forest dynamics

Often the evidence for resources controlling regeneration dynamics in forests, including Patagonian mixed forests [15, 29], has focused on the role of light. Until this study, the role of soil resources, versus light, had not been quantitatively investigated in South American *Nothofagus* forests. Our evidence suggests restricting soil nutrient depletion had a greater effect on HG than increased light in small canopy gaps (Table 3). Similarly, Coomes and Grubb [44] showed that increased soil resources better explained greater seedling growth in a very infertile Amazonian caatinga forest understory than canopy gap creation (i.e. increased light and potentially soil resources). Whereas, Lewis and Tanner [12] showed that canopy gap creation better explained increased seedling height growth in a Brazilian rainforest understory than trenching. We support a view that soil nutrient availability is relatively important to *Nothofagus* species that occur on infertile soils, have well-developed ectomycorrhizae, and dense root networks [45].

Regeneration dynamics in forest can reflect the traits of species and their plasticity in adjusting to variation in limiting resources [4, 46]. It was surprising that we found little evidence for difference in the responses of small seedlings of co-occurring species in relation to light and soil nutrient resources. As a consequence, we were unable to explain how a 50:50 ratio of seedlings of two species in the forest understory switched to heavy dominance by *N*. *betuloides* as trees. Our experiment imposed treatments within a diverse array of natural conditions [47]. It is possible that these conditions may have obscured any explanation because, for example, we did not account for the age of seedlings or the influence of microsites. However, the same resource responses by co-occurring *Nothofagus* species have also been found in New Zealand forests [48]. The expression of a trait might well be more complex. For example, Kunstler et al. [49] showed that light response differences among co-occurring species varied with the size of seedlings studied. Understanding how light, nutrients, and their interactions influence regeneration dynamics is now needed for a wider range of life-stages in forests.

## Supporting information

S1 Table Mean canopy openness with respect to canopy condition (CG = canopy gap, BUC = beneath undisturbed canopy) for the control and three treatments (C = control, F = fertilizer, T = root trenching, F+T = fertilizer+root trenching). All data used in publication.
(XLS)

S2 Table Mean initial density (D, seedlings m^-2^), initial root collar diameter (RCD, mm) and initial height (H, mm) of seedlings per plot by species (Nb = *Nothofagus betuloides*, Np = *Nothofagus pumilio*), canopy condition (CG = canopy gap, BUC = beneath undisturbed canopy) and treatment (C = control, F = fertilizer, T = root trenching, F+T = fertilizer+root trenching). All data used in publication.
(XLS)

S3 Table Mean absolute diameter growth (DG, mm), absolute height growth (HG, mm) of seedlings initial root collar diameter (RCD, mm) and initial height (H, mm) per plot by species (Nb = *Nothofagus betuloides*, Np = *Nothofagus pumilio*), canopy condition (CG = canopy gap, BUC = beneath undisturbed canopy) and treatment (C = control, F = fertilizer, T = root trenching, F+T = fertilizer+root trenching). All data used in publication.
(XLS)

S4 Table Percent seedling survival per plot by species (Nb = *Nothofagus betuloides*, Np = *Nothofagus pumilio*), canopy condition (CG = canopy gap, BUC = beneath undisturbed canopy) and treatment (C = control, F = fertilizer, T = root trenching, F+T = fertilizer+root trenching). All data used in publication.
(XLS)

S5 Table Mean above-ground biomass (A-GB, g), below-ground biomass (B-GB, g) and root to shoot ratio (R:S) of seedlings per plot by species (Nb = *Nothofagus betuloides*, Np = *Nothofagus pumilio*), canopy condition (CG = canopy gap, BUC = beneath undisturbed canopy) and treatment (C = control, F = fertilizer, T = root trenching, F+T = fertilizer+root trenching). All data used in publication.
(XLS)
